# Thermally controlling the singlet–triplet energy gap of a diradical in the solid state†
†Electronic supplementary information (ESI) available: Crystallographic data in CIF formats, powder EPR spectra, crystal structures and theoretical calculation. CCDC 1430382 and 1430383. For ESI and crystallographic data in CIF or other electronic format see DOI: 10.1039/c6sc01825d



**DOI:** 10.1039/c6sc01825d

**Published:** 2016-06-28

**Authors:** Yuanting Su, Xingyong Wang, Lei Wang, Zaichao Zhang, Xinping Wang, You Song, Philip P. Power

**Affiliations:** a State Key Laboratory of Coordination Chemistry , School of Chemistry and Chemical Engineering , Nanjing University , Nanjing 210093 , China . Email: xpwang@nju.edu.cn ; Email: yousong@nju.edu.cn; b School of Chemistry and Chemical Engineering , Huaiyin Normal University , Huai'an 223300 , China; c Department of Chemistry , University of California , Davis , CA 95616 , USA . Email: pppower@ucdavis.edu

## Abstract

Diradicals, molecules with two unpaired electrons, are reactive intermediates that play an important role in many fields. Their defining feature is the energy difference between their singlet and triplet states, which provides direct information on the extent of their electron exchange interactions. Such knowledge is essential for understanding their diradical character, which is controllable internally by modification of the electronic and steric properties of the substituents. We now report that the energy gap of a diradical in the solid state can also be controlled by an external stimulus. The dication diradical of 4,4′′-di(bisphenylamino)-*p*-terphenyl exhibits two singlet states with different exchange coupling constants at different temperatures as determined by SQUID and EPR measurements. The behavior is induced by the conformation change of the terphenyl bridge, the key structural unit of the species. The work presents an unprecedented instance of a thermally controllable singlet–triplet gap for a crystalline diradical and provides a novel diradical material relevant to the design of functional materials.

## Abstract 2

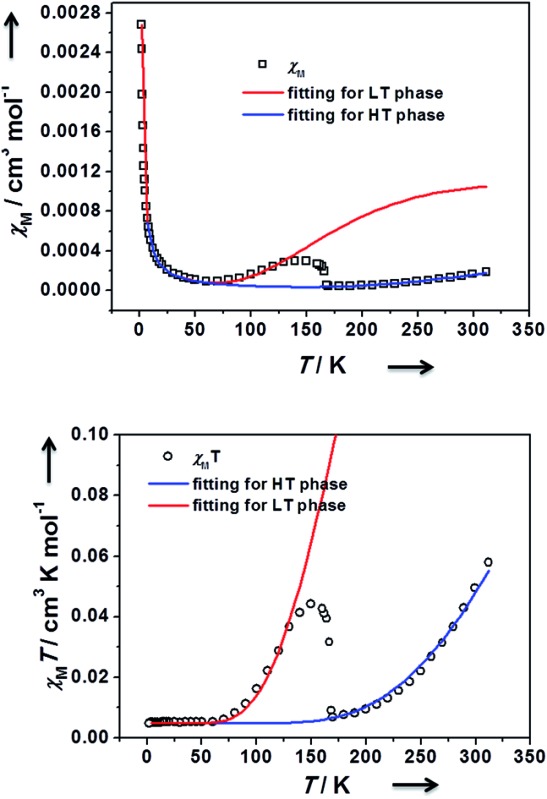
Diradicals, molecules with two unpaired electrons, are reactive intermediates that play an important role in many fields.

## Introduction

**Scheme 1:**
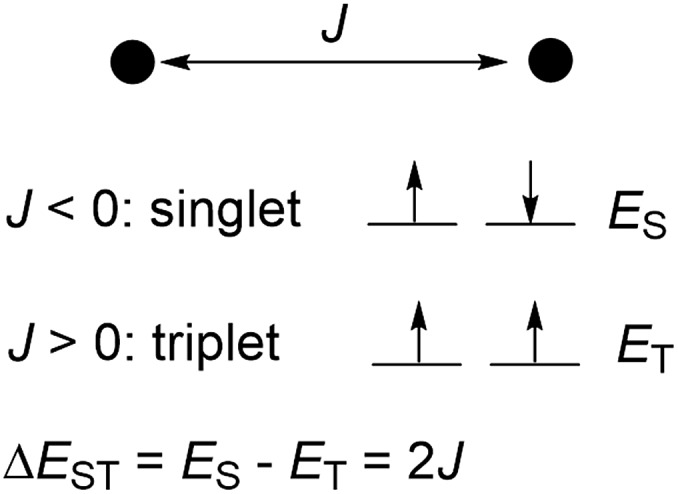
The electron exchange integral, electronic configurations and singlet–triplet energy gap of a diradical.

Diradicals,^1–15^ molecules with two unpaired electrons, are important reactive intermediates. They are important in the understanding the nature of chemical bonds in theoretical chemistry, and have fundamental significance in the fields in mechanistic chemistry, synthetic chemistry and biological chemistry. Because of their interesting optical, electronic and magnetic properties, diradicals also play important roles in materials chemistry expected to have promising applications as functional materials in quantum information processing systems, electronic devices, lithium ion batteries and organic spintronics. A diradical either has a singlet ground state (S, *S* = 0, spin multiplicity = 1) or has a triplet ground state (T, *S* = 1, spin multiplicity = 3), which is indicated by the negative or positive electron exchange interaction (*J*) between two unpaired electrons (Scheme 1). The singlet–triplet energy gap (Δ*E*_ST_ = *E*_S_ – *E*_T_ = 2*J*) provides direct information on the extent of the electron exchange interaction and is essential for understanding the diradical character. Estimation, determination and the control of the singlet–triplet energy gap have thus become the central themes in the study of diradicals.^1–10^ The use of different substituents and incorporation of heteroatoms in the structures have proven effective ways to alter the energy gap. However, control of Δ*E*_ST_ of a crystalline diradical by external stimuli (temperature, pressure, light, *etc.*) is highly desirable especially in the design of functional materials because no geometric modifications would be needed. Furthermore there is the possibility of reversibility by external stimuli. However, such phenomena have not been realized experimentally.

Recently, we and others have prepared a series of stable nitrogen-incorporating diradical dications,^16–24^ which can be viewed as analogues of intensively studied hydrocarbon diradicals such as Thiele's and Chichibabin's hydrocarbons.^25,26^ During the attempted synthesis of the analogue (**1^2+^**) of Müller's hydrocarbon,^27,28^ we observed that its Δ*E*_ST_ can be controlled by temperature—the first instance of a thermally controllable singlet–triplet gap for a diradical in the solid state.

## Results and discussion

**Scheme 2:**
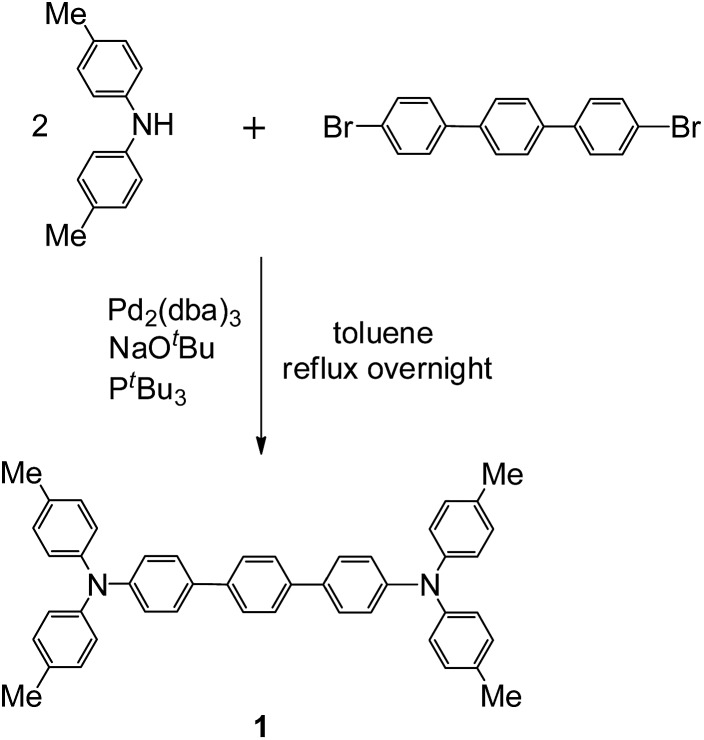
Synthesis of compound **1**.

Standard Buchwald–Hartwig aminations of 4,4′′-dibromo-*p*-terphenyl with di-*p*-tolylamine in the presence of sodium *tert*-butoxide in reflux toluene afforded compound **1** as white solid (Scheme 2). Upon oxidation with two equiv Ag[Al(OR_F_)_4_] (OR_F_ = OC(CF_3_)_3_)^29^ in CH_2_Cl_2_, the neutral precursor **1** was converted to dication **1^2+^** in a high 75% yield. The resultant dication is thermally stable as crystals under nitrogen or argon atmosphere and can be stored for several weeks at room temperature. Its solid geometry and electronic structure were investigated by single crystal X-ray diffraction, EPR, and SQUID measurements, in conjunction with DFT calculations.

**Fig. 1:**
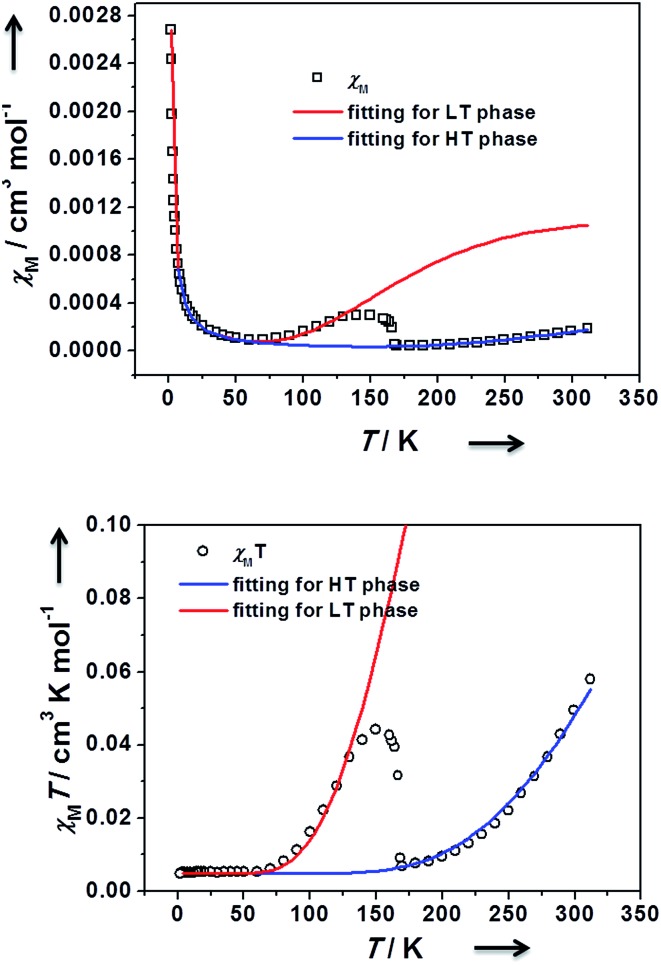
*χ* (above) and *χT* (below) *versus T* curves for the crystals of **1^2+^** in the SQUID measurements, and the fitting plots *via* the Bleaney–Bowers equation.

The temperature dependence of the magnetic susceptibility for a crystalline sample of **1^2+^**·2[Al(OR_F_)_4_]^–^ was investigated (Fig. 1). As the sample is cooled from room temperature, *χ*_M_ shows a slight decrease and becomes zero at 170 K. It then suddenly increases, up to a maximum at 130 K, followed by gradual decrease until zero at 50 K. The SQUID magnetic measurements suggest a phase transition with a wide thermal range from 130 to 170 K, exhibiting two singlet states. We repeated the process back and forth several times, and no hysteresis loop was observed. The magnetic susceptibilities with a phase transition can be well fitted with the Bleaney–Bowers equation.^30^ The singlet–triplet gaps (or intramolecular exchange coupling constants) were estimated to be 2*J* = –400.57 cm^–1^ for the low temperature (LT) phase and –872.84 cm^–1^ for the high temperature (HT) phase, respectively, indicating that **1^2+^** has a singlet state for each phase, both of which can be thermally excited to their triplet excited states.

**Fig. 2:**
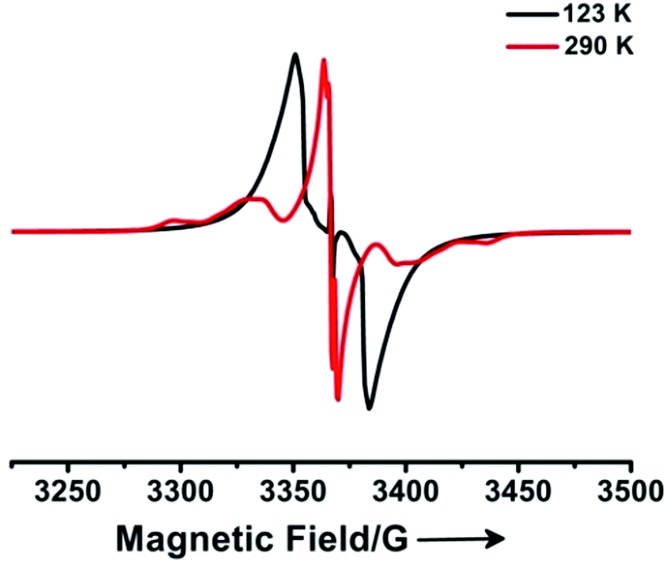
The powder EPR spectra of **1^2+^** at 290 and 123 K.

The magnetic behavior of **1^2+^** was confirmed by means of electron paramagnetic resonance spectroscopy. The powder EPR spectra of **1^2+^**·2[Al(OR_F_)_4_]^–^ display featureless broad signals with two different *g* factors for LT (2.00259) and HT phases (2.00213), respectively (Fig. 2 and S1 and S2†), which agree well with the corresponding critical temperatures determined in the SQUID measurements. The forbidden Δ*m*_s_ = ±2 half-field absorptions were also observed (Fig. S3–S5†). The broaden signals are attributed to spin-triplet species, which together with the forbidden resonances resulting from Δ*m*_s_ = ±2 transitions and SQUID measurements unambiguously indicate that **1^2+^** exists as a singlet diradical with thermal population of its higher energy triplet state for both LT and HT phases.

**Fig. 3:**
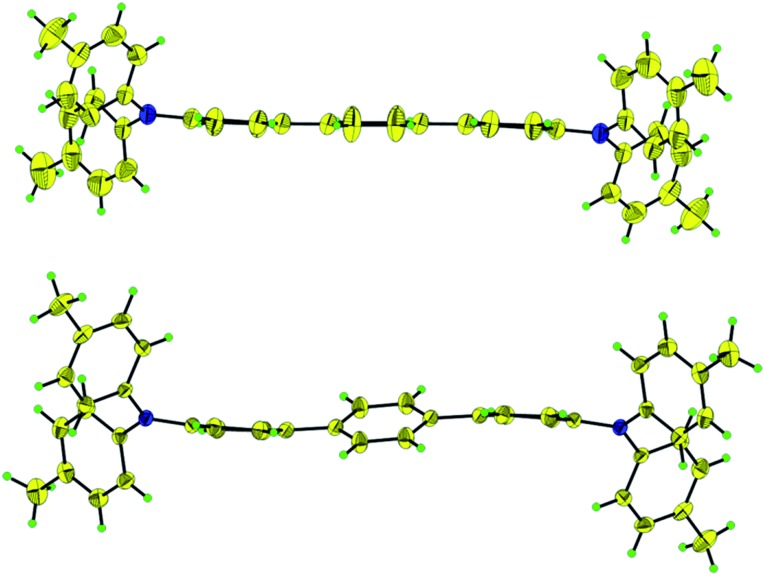
Thermal ellipsoid (50%) drawings of **1^2+^** for HT (top) and LT (below) phases. Blue, nitrogen; yellow, carbon; green, hydrogen.

To rationalize the magnetic behavior, the structure of **1^2+^** was examined by single crystal X-ray diffraction at 123 (LT) and 200 K (HT), respectively. Crystals suitable for X-ray crystallographic studies were obtained by cooling the solution of salt **1^2+^**·2[Al(OR_F_)_4_]^–^ in CH_2_Cl_2_. The structures of dication **1^2+^** at LT and HT are illustrated as stereoviews in Fig. 3 and their important structural parameters are given in Table 1 and Fig. S6 and S7.† There is a crucial difference between molecular structures. The terphenyl backbone of **1^2+^** is sigmoidal with a large torsion angle (*θ* = 26.76°) at LT while it is nearly planar (*θ* = 3.78°) at HT. At LT the length (1.468(3) Å) of bond *k* between the two neighboring phenyl rings of the terphenyl bridge is shorter than that of a typical biphenyl single bond (1.48 Å), but longer than that of Chichibabin's hydrocarbon (1.448(4) Å),^26^ indicating that there is considerable diradical character for the LT phase. In contrast the length of bond *k* (1.446(5) Å) is shorter at HT, which together with the stronger quinoidal terphenyl bridge is consistent with the stronger exchange coupling for the HT phase shown by SQUID measurements. Reduced bond length alternation (BLA) of a bridged phenyl ring has been considered as a signature of diradical character in hydrocarbon diradicals,^16–26^ which is the difference between longitudinal bonds (*e.g.* bonds *h* and *i* in B, Table 1) and transverse bonds (*e.g.* bond *j* in B) of one phenyl ring of the terphenyl bridge in this work. BLAs for phenyl rings A and B, abbreviated as BLA_A_ and BLA_B_, for the LT phase (0.031 and 0.023) are much less than that of Chichibabin's hydrocarbon (0.052),^26^ supporting the strong diradical character of **1^2+^** at LT phase. At HT, BLA_B_ (0.022) is similar while BLA_A_ (0.053) is much higher, consistent with the weaker diradical property of **1^2+^** for the HT phase. The molecular packings for the LT and HT phases are almost the same, in which dications of **1^2+^** are separated by [Al(OR_F_)_4_]^–^ anions through F···H hydrogen bonding. Each terminal phenyl ring of terphenyl backbone connects with the counter anions by three F···H hydrogen bonding, one of them almost does not change (2.634 Å (HT), 2.637 Å (LT)) while other two shorten from HT (2.588, 2.562 Å) to LT (2.409, 2.494 Å), which may also contribute to the geometry change of the terphenyl backbone from nearly planar at HT to sigmoidal with a large torsion angle at LT.

**Table 1:** Selected experimental and calculated bond lengths (Å), torsion angle (°) and diradical character (*y*) for **1^2+^**

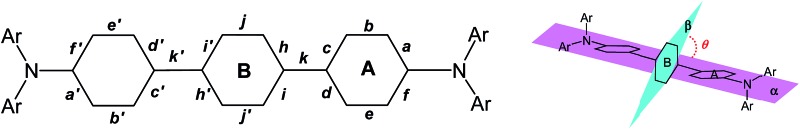											
	Avg N–Ar	Avg N–C_terphenyl_	Bond *k*	Avg *a*, *c*, *d*, and *f*	Avg *b* and *e*	BLA_A_ ^*b*^	Avg *h* and *i*	Bond *j*	BLA_B_ ^*b*^	*θ*	*y* ^*c*^
X-ray (200 K)	1.430(5)	1.366(4)	1.446(5)	1.415(5)	1.362(5)	0.053	1.396(6)	1.374(6)	0.022	3.78	0.79
CS^*a*^ ^,^ ^*d*^	1.424	1.383	1.451	1.422	1.375	0.047	1.422	1.377	0.045	17.38	—
X-ray (123 K)	1.414(3)	1.392(3)	1.468(3)	1.407(3)	1.376(3)	0.031	1.402(3)	1.379(3)	0.023	26.76	0.89
OS^*a*^ ^,^ ^*d*^	1.414	1.411	1.477	1.411	1.385	0.026	1.409	1.388	0.021	32.17	0.93
T^*a*^ ^,^ ^*b*^	1.413	1.415	1.479	1.410	1.386	0.024	1.407	1.389	0.018	34.06	—

^*a*^CS = closed-shell singlet, OS = open-shell singlet, T = triplet.

^*b*^BLA = bond length alteration, *i.e.* BLA_A_ is the difference between the average of length of longitudinal bonds (*a*, *c*, *d* and *f*) and the average of length of transverse bonds (*b* and *e*) in ring A, BLA_B_ is the difference between the average of *h* and *i* bond lengths and *j* bond length in ring B.

^*c*^Calculated at the level of UBH and HLYP/6-31G(d).

^*d*^Calculated at the level of (U)B3LYP/6-31G(d).

**Fig. 4:**
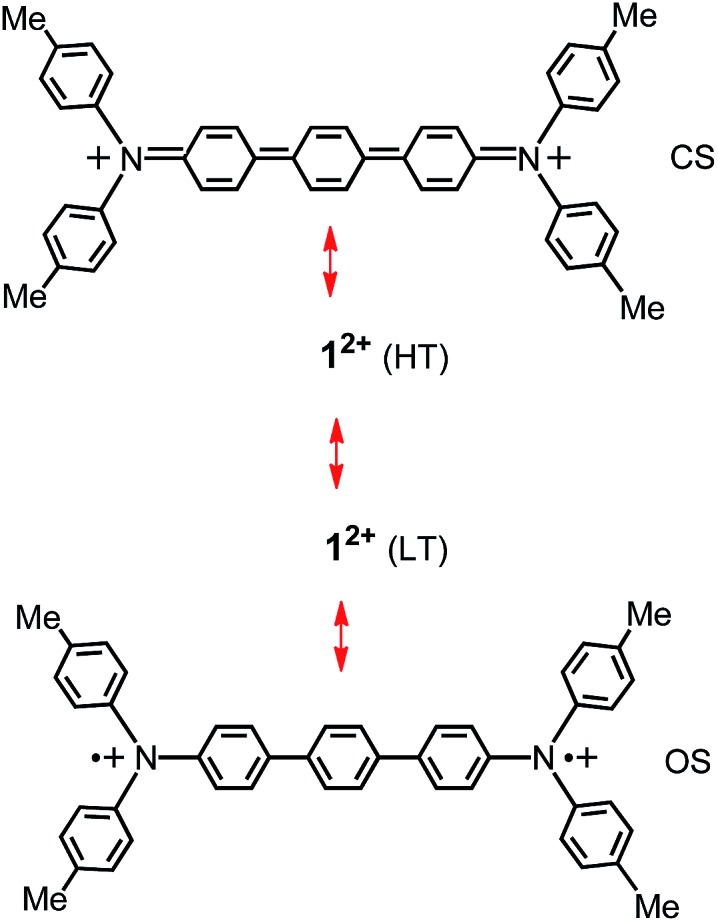
Resonance structures of **1^2+^**.

To further understand the electronic structures, we carried out DFT calculations for species **1^2+^**.^31^ Full geometry optimizations were performed at the (U)B3LYP/6-31G(d) level and the obtained stationary points were characterized by frequency calculations. Bond lengths and their BLAs in the averages of bonds of the optimized closed-shell singlet (CS), open-shell singlet (OS) and pure diradical triplet (T), as well as diradical character *y*, are listed in Table 1. The structural parameters of **1^2+^** and BLAs at LT are very close to those of OS. At HT, all other parameters are similar to those of CS, except for those of the central phenyl ring, where bonds *h* and *i* are significantly shorter than those of CS. Correspondingly the BLA_B_ (0.022) is quite smaller than that (0.045) of CS, indicating there is still some diradical character for **1^2+^** at HT. The diradical character *y*, estimated by the occupancy of the lowest unoccupied natural orbital (LUNO), represents the “degree” of the singlet diradical character.^20–24^ The *y* value (0.89) of the X-ray structure of **1^2+^** at LT is larger than that (0.79) at HT, but both of which are close to that (0.93) of OS, calculated at the UBH and HLYP/6-31G(d) level. The above discussion based on comparison of bond lengths, BLAs and *y* values clearly shows singlet states with intermediate diradical character for **1^2+^**. Dication **1^2+^** is best described as a resonance hybrid of diradical structure and quinoidal structure (Fig. 4), with a greater tendency to diradical structure for the LT phase.

## Conclusions

The preparation and characterization of 4,4′′-di(bisphenylamino)-*p*-terphenyl dication diradicaloid (**1^2+^**), the nitrogen analogue of Müller's hydrocarbon, has shown that it exhibits two singlet states at different temperature stages as indicated by EPR spectroscopy and SQUID measurements. A bond-length and torsion angle analysis conducted on the basis of single crystal X-ray diffraction at high and low temperatures indicates that the difference in the conformation of the terphenyl bridge, the key structural unit of **1^2+^**, is responsible for the existence of the two singlet states. The interconversion is induced by intramolecular interaction, which was only previously observed in the solution for a diradical.^32–35^ The phenomenon of thermally controllable singlet–triplet gap is unprecedented for a crystalline diradical, and the work represents a significant step forward towards a better understanding of diradicals. In addition, it provides a novel diradical material for use in the design of functional materials. Further studies on bis(triarylamine) dications with various combinations of bridge types as well as investigation of the application of this unique dication system to functional materials are under way.

## Experimental section

### General experimental procedures

All manipulations were carried out under an Ar or N_2_ atmosphere by using standard Schlenk or glove box techniques. Solvents were dried prior to use. 4,4′′-Dibromo-*p*-terphenyl and di-*p*-tolylamine were purchased from Alfa Aesar and used upon arrival. Ag[Al(OR_F_)_4_] was prepared by the published procedure.^29^ The ^1^H NMR and ^13^C NMR spectra were recorded in solution of CDCl_3_ on a Bruker DRX 500 NMR spectrometer with tetramethylsilane (TMS) as the internal standard. UV/Vis spectra were recorded on the Lambda 750 spectrometer. MALDI-TOF mass spectra (MS) were recorded on a Bruker Autoflex instrument. Element analyses were performed at Shanghai Institute of Organic Chemistry, the Chinese Academy of Sciences. EPR spectra were obtained using Bruker EMX-10/12 X-band variable-temperature apparatus. Magnetic measurements were performed using a Quantum Design MPMS XL-7 SQUID magnetometer in the temperature range 5–350 K with field up to 7 T. Crystal of **1^2+^**·2[Al(OR_F_)_4_]^–^ was removed from a Schlenk flask under N_2_ and immediately covered with a layer of polyisobutylene oil. A suitable crystal was selected, attached to a glass fiber on a copper pin, and quickly placed in the cold N_2_ stream of the diffractometer. X-ray data were collected at 123 K and 200 K, respectively, on a Bruker APEX DUO CCD diffractometer with Mo Kα (*λ* = 0.71073 Å) radiation. Integrations were performed with SAINT. Absorption corrections were applied using SADABS program. The crystal structures were solved by direct methods and refined by full matrix least-squares based on *F* ^2^ using the SHELXTL program. All nonhydrogen atoms were refined anisotropically. Details of the data collections and refinements are given in Table S1.†

#### Synthesis of neutral **1**

A mixture of 4,4′′-dibromo-*p*-terphenyl (970 mg, 2.50 mmol), di-*p*-tolylamine (1.08 g, 5.50 mmol), sodium *tert*-butoxide (480 mg, 5.00 mmol), Pd_2_(dba)_3_ (18.3 mg, 20 μmol) and P^*t*^Bu_3_ (0.10 mL of a 10% solution in hexane, 19.2 μmol) in toluene (30 mL) was stirred under nitrogen atmosphere at 110 °C overnight. The solvent was reduced *in vacuo*. The residue was dissolved in dichloromethane and washed with water. The organic layer was dried over MgSO_4_ and the solvent was removed. The crude product was precipitated into rapidly stirring methanol (50 mL), collected by filtration and washed with cold methanol to yield fine white powders (1.38 g, 89%). ^1^H NMR (500 MHz, CDCl_3_): *δ* 7.61 (s, 4H), 7.48 (d, *J* = 8.6 Hz, 4H), 7.07 (m, 20H), 2.33 (s, 12H). ^13^C NMR (125 MHz, CDCl_3_): 147.89, 145.57, 139.28, 133.98, 132.90, 130.24, 127.72, 127.12, 125.03, 123.03, 21.17. HRMS (MALDI-TOF, *m*/*z*): calcd for C_46_H_40_N_2_, 620.319; found, 620.350.

#### Synthesis of dication salt **1^2+^**·2[Al(OR_F_)_4_]^–^

Under anaerobic and anhydrous conditions, a mixture of **1** (0.124 g, 0.20 mmol) and Ag[Al(OR_F_)_4_] (0.451 g, 0.42 mmol) in CH_2_Cl_2_ was stirred at room temperature overnight. The resultant solution was filtered to remove the gray precipitate (Ag metal). The filtrate was then concentrated and stored at around –30 °C for 1 day to afford blue-green crystals of **1^2+^**·2[Al(OR_F_)_4_]^–^. Yield: 0.383 g, 75%; UV-Vis (CH_2_Cl_2_): *λ*_max_ = 875, 644 (shoulder) nm; elemental analysis (%) calcd: C, 36.67; H, 1.58; N, 1.10; found: C 36.96, H 1.47, N 1.15.

### Computational details

All calculations were performed with the Gaussian 09 program suite.^31^ The symmetry-broken approach was applied for open-shell singlet calculations. All the geometry optimizations were carried out at the (U)B3LYP/6-31G(d) level of theory. The obtained stationary points were characterized by frequency calculations. The diradical character (*y*) was obtained from the occupation number of the lowest unoccupied natural orbital (LUNO) at the UBH and HLYP/6-31G(d) level.
