# Supplementary material for: Thermally controlling the singlet–triplet energy gap of a diradical in the solid state
Source: Chem Sci. 2016 Jun 28;7(10):6514–8. doi: 10.1039/c6sc01825d (PMC5125349; doi:10.1039/c6sc01825d)
Supplement: Supplementary file 1 [file SC-007-C6SC01825D-s001.pdf]

Electronic Supplementary Material (ESI) for Chemical Science.
This journal is © The Royal Society of Chemistry 2016

Supporting Information for

Thermally Controlling the Singlet-Triplet Energy Gap of a Diradical in the Solid State

Yuanting Su, Xingyong Wang, Lei Wang, Zaichao Zhang, Xinping Wang,* You Song* and Philip P. Power*

Contents

Table S1. Crystal data and structure refinement... ....S2

Figure S1. Powder EPR spectra of **1**$^{2+}$•2[Al(OR$_F$)$_4$]$^-$ at various temperatures (120-170K)..............................S3

Figure S2. Powder EPR spectra of **1**$^{2+}$•2[Al(OR$_F$)$_4$]$^-$ at various temperatures (190-290K)... ....S4

Figure S3. Powder EPR spectrum of **1**$^{2+}$•2[Al(OR$_F$)$_4$]$^-$ at 123 K...S5

Figure S4. Powder EPR spectrum of **1**$^{2+}$•2[Al(OR$_F$)$_4$]$^-$ at 200 K..... ....S6

Figure S5. Powder EPR spectrum of **1**$^{2+}$•2[Al(OR$_F$)$_4$]$^-$ at 290 K..... ....S7

Figure S6. Packing diagram of **1**$^{2+}$ at 200 K..S8

Figure S7. Packing diagram of **1**$^{2+}$ at 123 K..S9

Coordinates for calculated geometries S10

Table S1. Crystal data and structure refinement for **1**$^{2+}$•2[Al(OR$_F$)$_4$]$^-$

	123 K	200 K
formula	$C_{78}H_{40}Al_2F_{72}N_2O_8$	$C_{78}H_{40}Al_2F_{72}N_2O_8$
Mr [g mol^{-1}]	2555.08	2555.08
crystal system	Monoclinic	Monoclinic
space group	*P*2(1)/c	*P*2(1)/c
Z	2	2
Temp. (K)	123(2)	200(2)
μ(mm^{-1})	0.233	0.226
a (Å)	17.262(3)	17.4037(17)
b (Å)	13.956(2)	14.1343(13)
c (Å)	20.301(3)	20.914(2)
α (°)		
β (°)	109.105(3)	111.7830(15)
γ (°)		
V [Å^3]	4621.4(13)	4777.2(8)
*R*1 (*I*>2σ(*I*))	0.0489	0.0905
*wR*2 (all data)	0.1394	0.2169

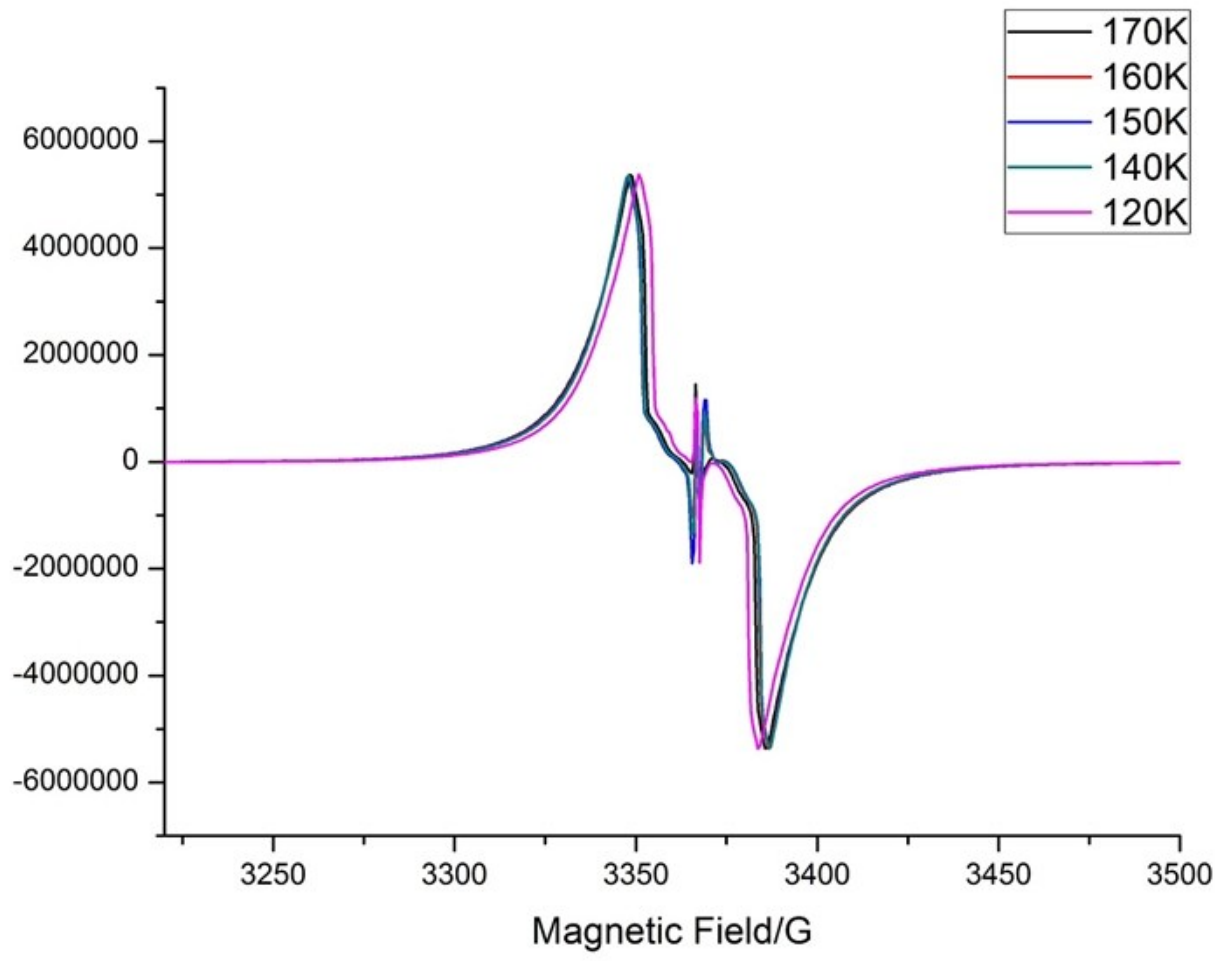

Figure S1. Powder EPR spectra of $\mathbf{1}^{2+}$•2[Al(OR$_F$)$_4$]$^-$ at various temperatures (120 – 170 K). The central peak shows the signal derived from the mono radical impurity.

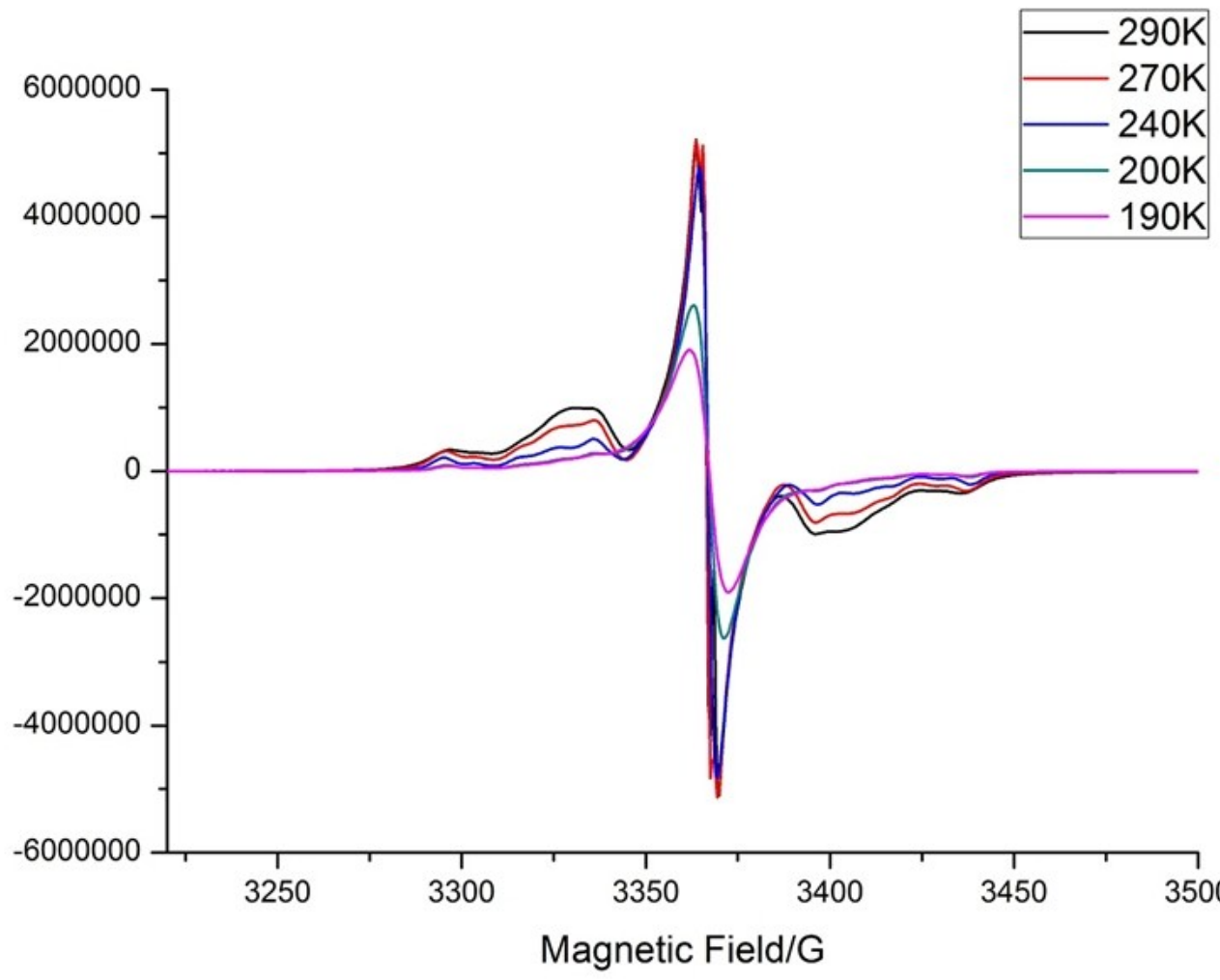

Figure S2. Powder EPR spectra of $\mathbf{1}^{2+}$•2[Al(OR$_F$)$_4$]$^-$ at various temperatures (190 – 290 K). The central peak shows the signal derived from the mono radical impurity.

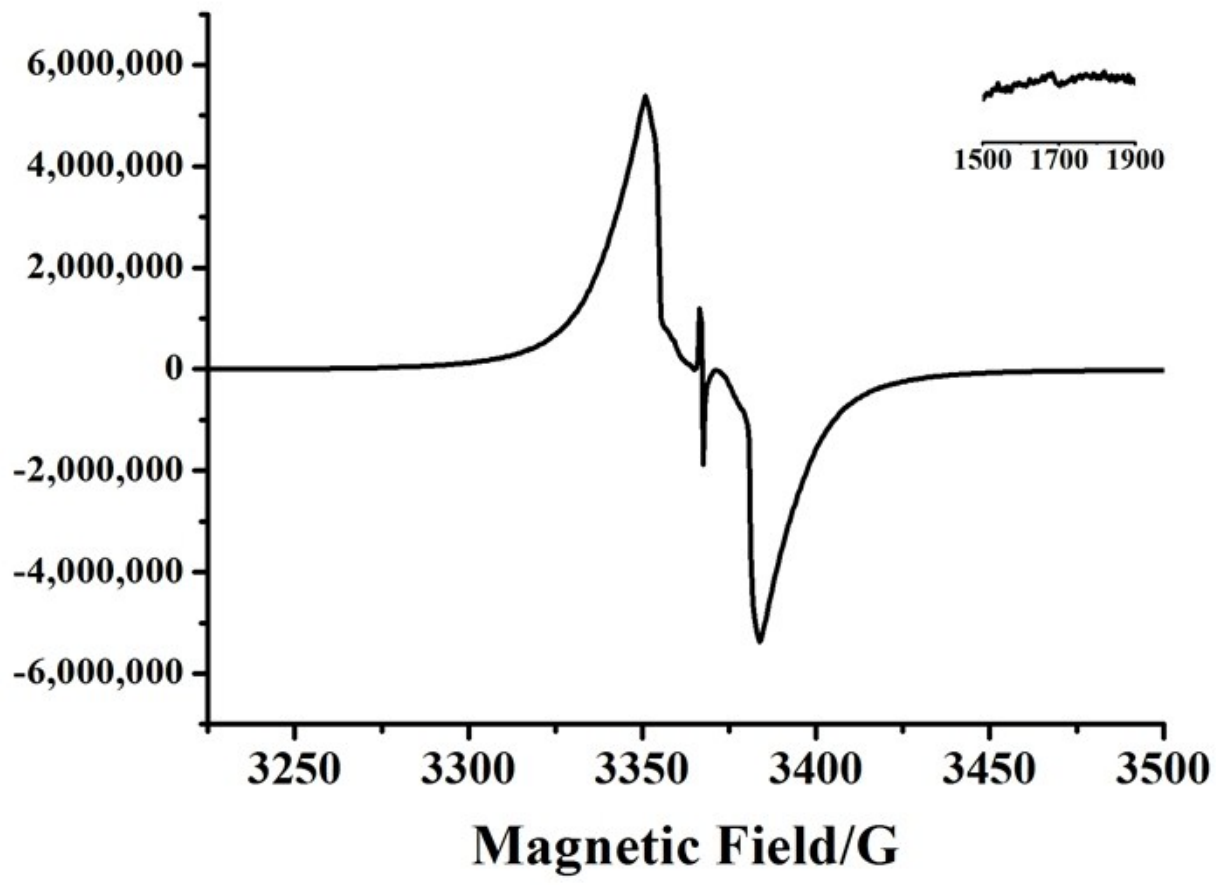

Figure S3. Powder EPR spectra of $\mathbf{1}^{2+}$•2$[Al(OR_F)_4]^-$ at 123K. The central peak shows the signal derived from the mono radical impurity.

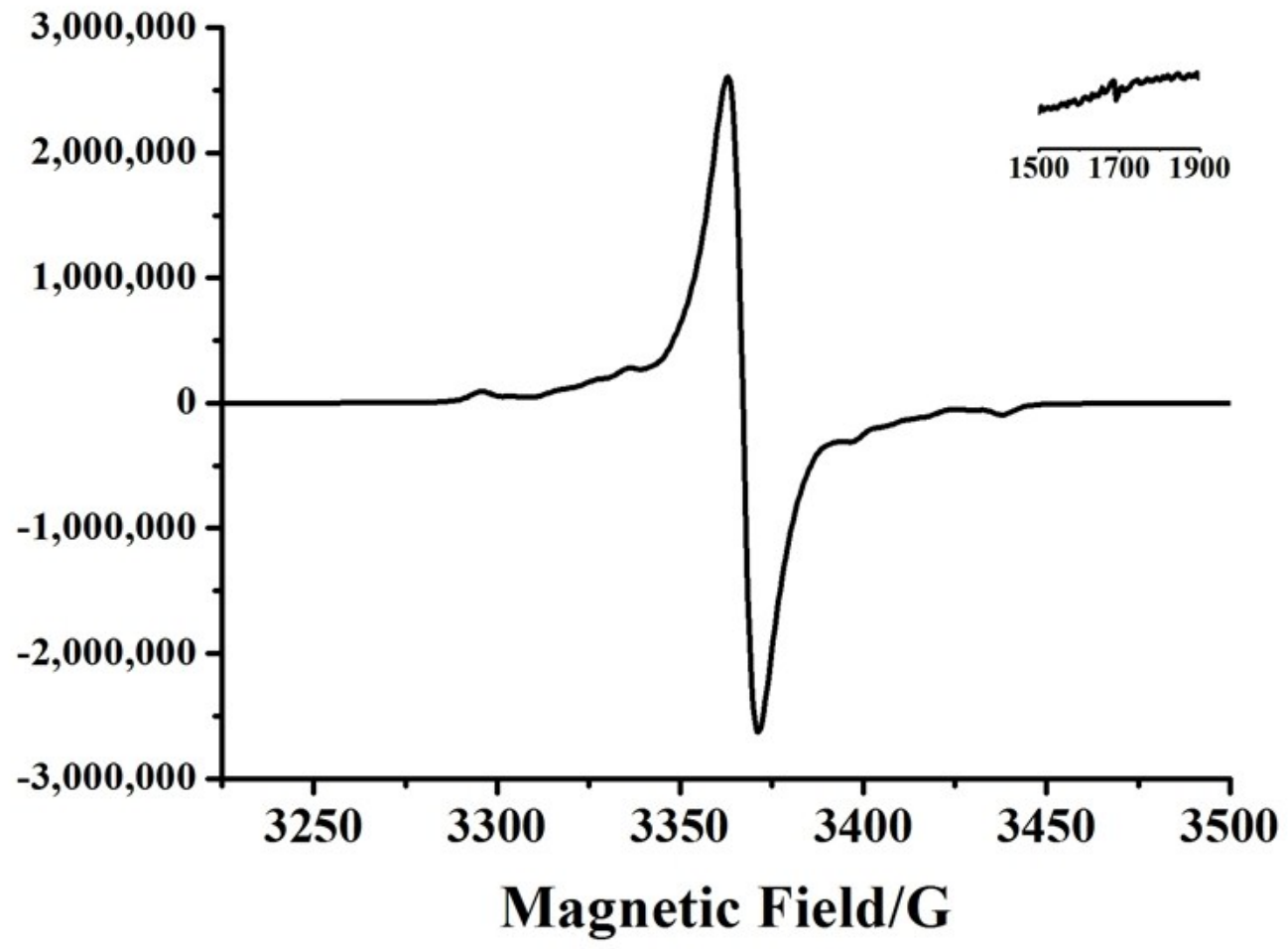

Figure S4. Powder EPR spectra of $\mathbf{1}^{2+}$•2$[Al(OR_F)_4]^-$ at 200K.

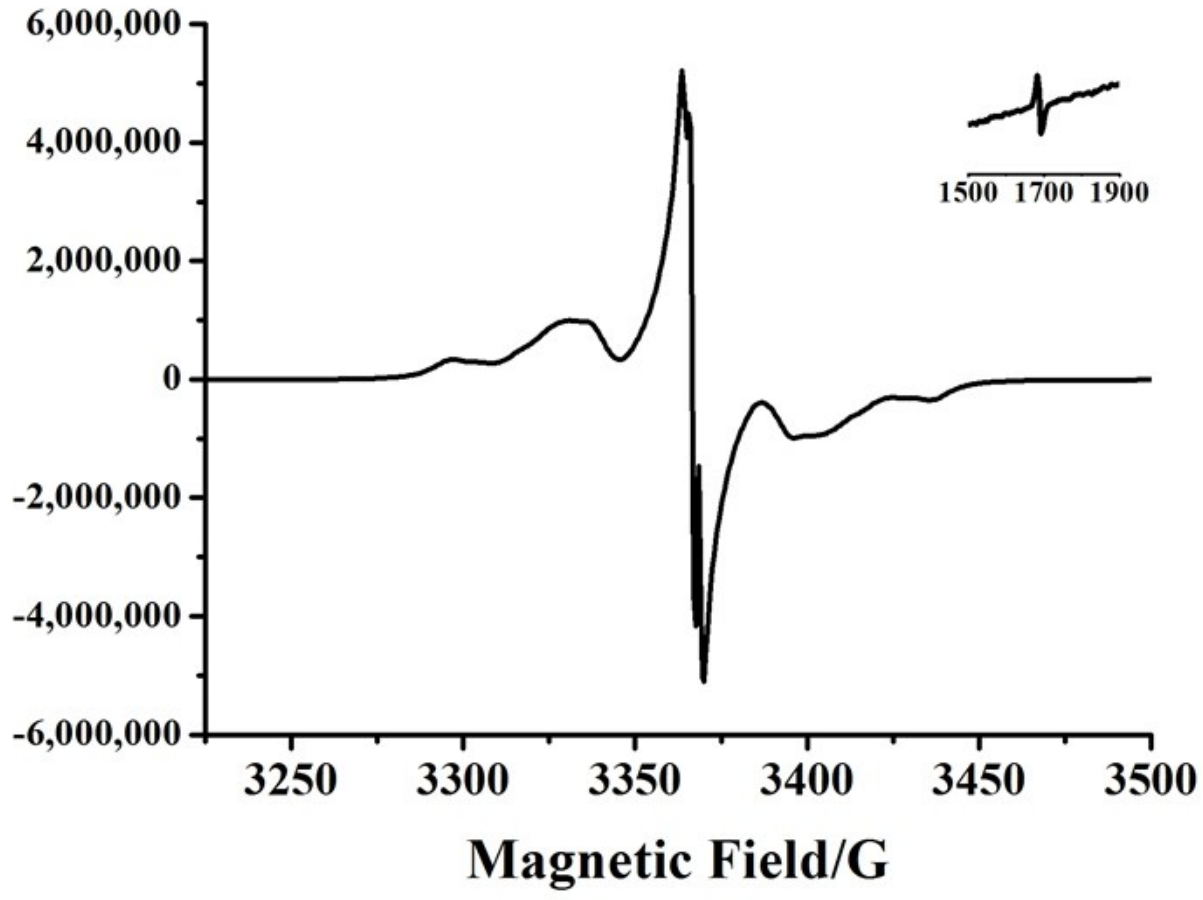

Figure S5. Powder EPR spectra of $\mathbf{1}^{2+}$•2[Al(OR$_F$)$_4$]$^-$ at 290K.

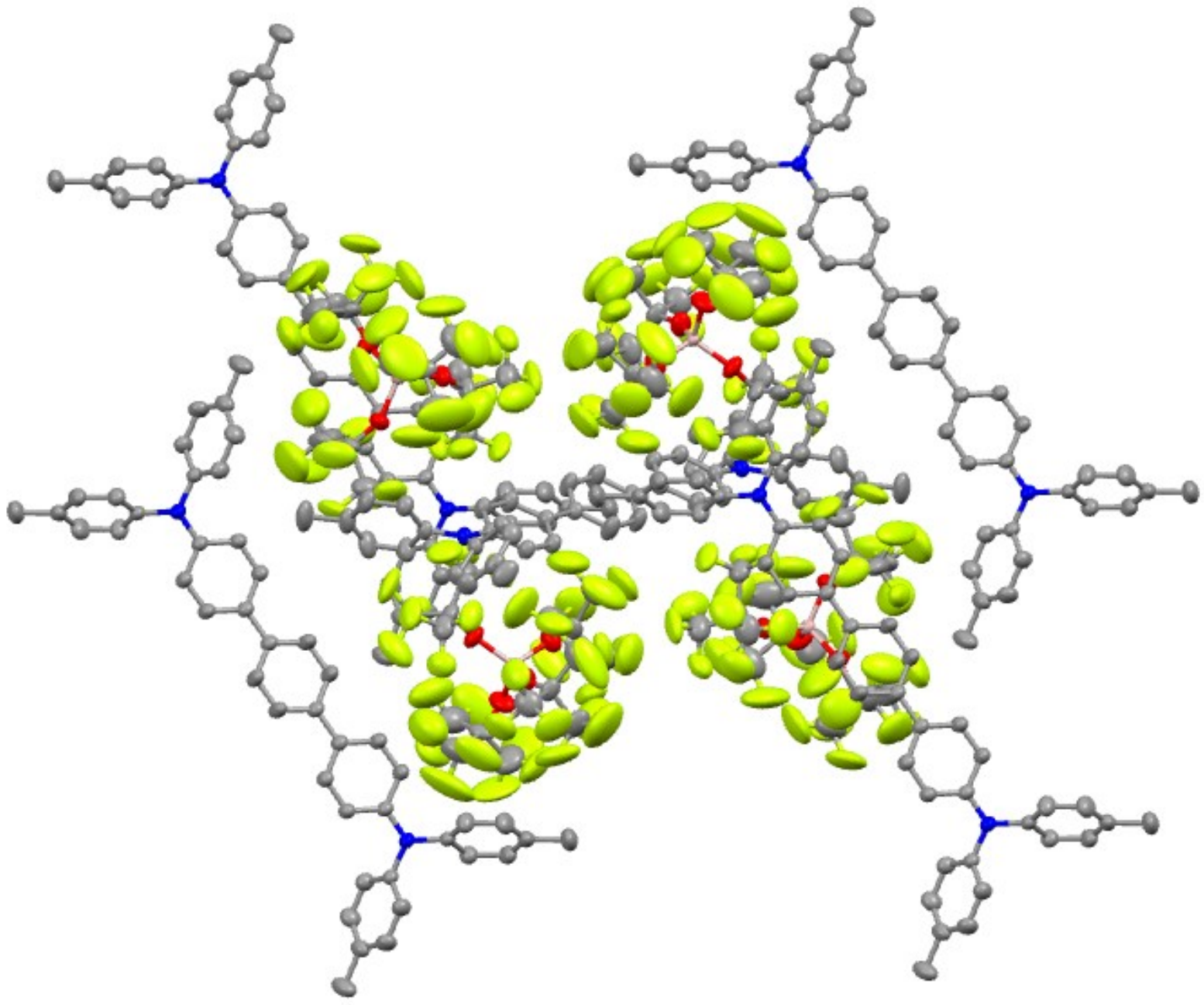

Figure S6. Packing diagram of $\mathbf{1}^{2+}$•2[Al(OR$_F$)$_4$]$^-$ at 200 K.

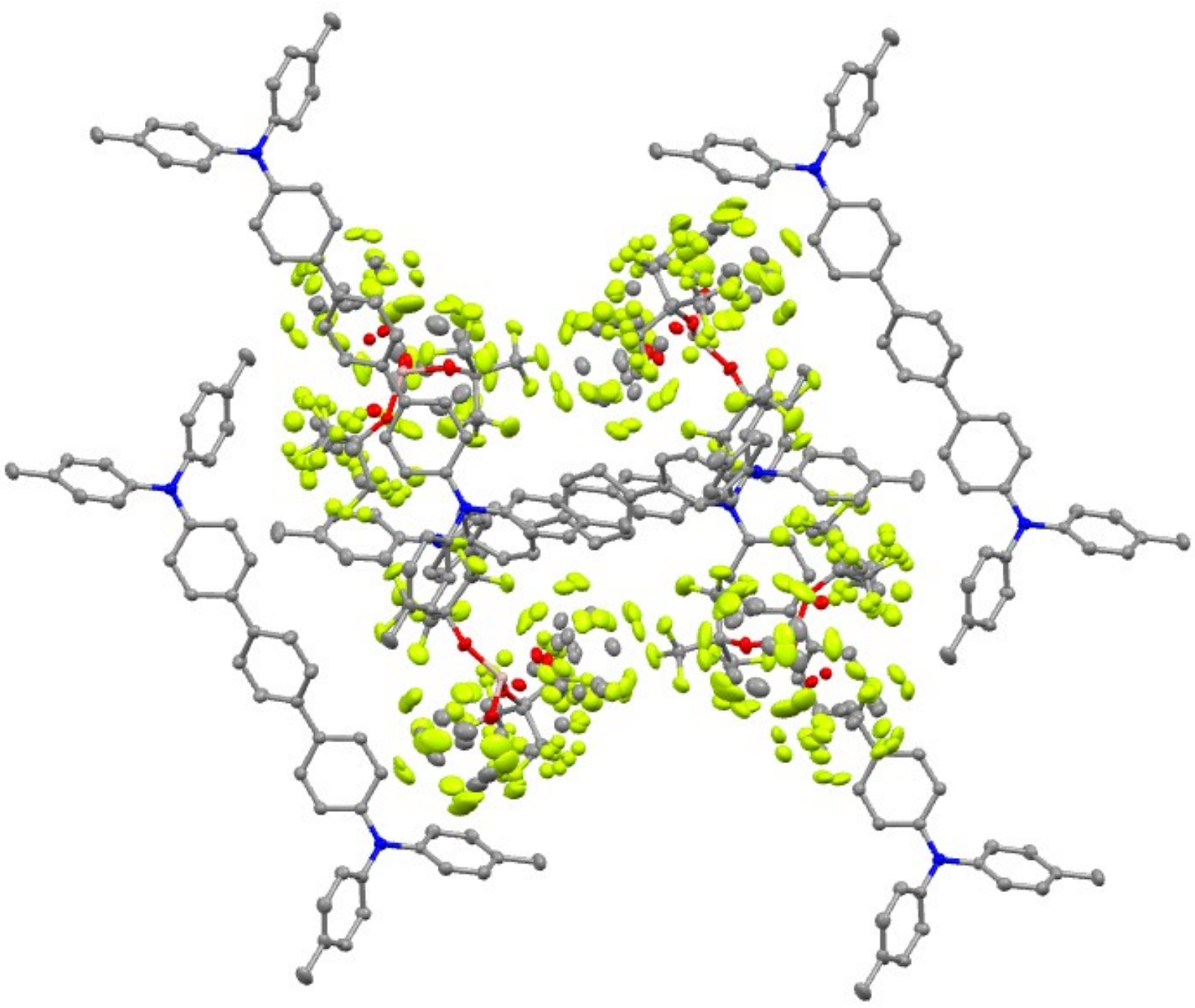

Figure S7. Packing diagram of $\mathbf{1}^{2+}$•2[Al(OR$_F$)$_4$]$^-$ at 123 K.

Coordinates for calculated geometries:

1^{2+}-CS

C	-1.437688000	0.000040000	0.000246000
C	-0.688524000	-0.834357000	-0.874442000
H	-1.199005000	-1.457558000	-1.599616000
C	0.688108000	-0.834411000	-0.874681000
H	1.198288000	-1.457657000	-1.600027000
C	-5.742726000	0.000159000	0.000966000
C	-5.012218000	-1.071003000	-0.583277000
H	-5.545986000	-1.887754000	-1.054426000
C	-3.637104000	-1.055587000	-0.592243000
H	-3.123536000	-1.890520000	-1.054472000
C	-2.888662000	0.000086000	0.000501000
C	-3.636858000	1.055794000	0.593498000
H	-3.123114000	1.890673000	1.055629000
C	-5.011979000	1.071303000	0.584964000
H	-5.545527000	1.888023000	1.056437000
C	-7.857783000	1.221521000	-0.002897000
C	-8.977196000	1.370130000	0.836472000
H	-9.258791000	0.567403000	1.509856000
C	-9.692735000	2.558491000	0.822243000
H	-10.543508000	2.672087000	1.488177000
C	-9.333533000	3.621877000	-0.027172000
C	-8.216200000	3.451334000	-0.863630000
H	-7.929882000	4.251073000	-1.540740000
C	-7.482986000	2.272080000	-0.859868000
H	-6.643707000	2.146581000	-1.536525000
C	-10.142354000	4.890634000	-0.058272000
H	-11.022718000	4.769845000	-0.703797000
H	-9.562218000	5.730900000	-0.449861000
H	-10.509302000	5.155909000	0.938331000
C	-7.857315000	-1.221576000	0.006120000
C	-7.482871000	-2.270871000	0.864285000
H	-6.645269000	-2.143677000	1.542699000
C	-8.218976000	-3.448744000	0.872514000
H	-7.936207000	-4.245255000	1.554733000
C	-9.335950000	-3.620238000	0.036518000
C	-9.698315000	-2.555455000	-0.810511000
H	-10.555394000	-2.665749000	-1.469045000
C	-8.980098000	-1.369188000	-0.829423000
H	-9.265466000	-0.564813000	-1.499211000
C	-10.119622000	-4.905068000	0.034147000

H	-9.880746000	-5.501799000	-0.856253000
H	-11.197791000	-4.714459000	0.010509000
H	-9.896621000	-5.516974000	0.912282000
N	-7.125559000	0.000110000	0.001395000
C	1.437635000	-0.000068000	-0.000249000
C	0.688466000	0.834327000	0.874441000
H	1.198938000	1.457532000	1.599617000
C	-0.688166000	0.834385000	0.874676000
H	-1.198354000	1.457628000	1.600021000
C	5.742691000	-0.000189000	-0.000962000
C	5.012168000	1.070900000	0.583409000
H	5.545928000	1.887591000	1.054670000
C	3.637059000	1.055483000	0.592369000
H	3.123503000	1.890361000	1.054707000
C	2.888607000	-0.000114000	-0.000503000
C	3.636814000	-1.055743000	-0.593634000
H	3.123081000	-1.890560000	-1.055888000
C	5.011930000	-1.071253000	-0.585104000
H	5.545469000	-1.887905000	-1.056704000
C	7.857784000	-1.221522000	0.002697000
C	8.977128000	-1.369983000	-0.836786000
H	9.258625000	-0.567172000	-1.510110000
C	9.692729000	-2.558310000	-0.822746000
H	10.543447000	-2.671792000	-1.488772000
C	9.333664000	-3.621801000	0.026594000
C	8.216403000	-3.451402000	0.863175000
H	7.930190000	-4.251221000	1.540234000
C	7.483127000	-2.272184000	0.859600000
H	6.643910000	-2.146798000	1.536355000
C	10.142560000	-4.890517000	0.057490000
H	11.023038000	-4.769707000	0.702853000
H	9.562543000	-5.730831000	0.449148000
H	10.509336000	-5.155729000	-0.939194000
C	7.857270000	1.221569000	-0.005915000
C	7.482901000	2.270956000	-0.863994000
H	6.645352000	2.143844000	-1.542488000
C	8.219017000	3.448827000	-0.872036000
H	7.936306000	4.245415000	-1.554190000
C	9.335924000	3.620221000	-0.035935000
C	9.698209000	2.555345000	0.811012000
H	10.555233000	2.665561000	1.469633000
C	8.979985000	1.369079000	0.829734000
H	9.265296000	0.564629000	1.499457000

C	10.119634000	4.905029000	-0.033383000
H	9.881087000	5.501463000	0.857302000
H	11.197805000	4.714368000	-0.010169000
H	9.896371000	5.517236000	-0.911240000
N	7.125513000	-0.000128000	-0.001391000

1^{2+}-OS

N	-7.139505000	-0.000015000	0.000071000
C	-5.728082000	-0.000057000	0.000101000
C	-5.015373000	-0.965918000	-0.739989000
H	-5.553813000	-1.701651000	-1.327085000
C	-3.630282000	-0.955057000	-0.739414000
H	-3.102735000	-1.712493000	-1.309643000
C	-2.899674000	-0.000074000	0.000093000
C	-3.630266000	0.954908000	0.739616000
H	-3.102707000	1.712341000	1.309838000
C	-5.015357000	0.965787000	0.740197000
H	-5.553782000	1.701534000	1.327287000
C	-1.422886000	-0.000062000	0.000066000
C	-0.694069000	0.416631000	1.131192000
H	-1.217845000	0.703872000	2.037584000
C	0.694107000	0.416656000	1.131160000
H	1.217914000	0.703915000	2.037530000
C	-7.849238000	-1.222946000	0.009221000
C	-7.393599000	-2.304376000	0.790634000
H	-6.511254000	-2.189507000	1.411160000
C	-8.107385000	-3.493203000	0.801473000
H	-7.763201000	-4.311567000	1.427287000
C	-9.278914000	-3.653990000	0.037896000
C	-9.717625000	-2.564229000	-0.738994000
H	-10.612688000	-2.668092000	-1.345792000
C	-9.025143000	-1.364026000	-0.757389000
H	-9.365233000	-0.544453000	-1.381184000
C	-10.031268000	-4.956469000	0.032898000
H	-9.749847000	-5.559265000	-0.841251000
H	-11.112314000	-4.794659000	-0.023558000
H	-9.815082000	-5.551326000	0.924642000
C	-7.849130000	1.222983000	-0.009181000
C	-9.025079000	1.364234000	0.757411000
H	-9.365247000	0.544729000	1.381254000

C	-9.717383000	2.564479000	0.738949000
H	-10.612427000	2.668503000	1.345763000
C	-9.278521000	3.654188000	-0.038052000
C	-8.107007000	3.493257000	-0.801493000
H	-7.762651000	4.311565000	-1.427267000
C	-7.393341000	2.304296000	-0.790583000
H	-6.510956000	2.189337000	-1.411035000
C	-10.031297000	4.956428000	-0.033240000
H	-9.761608000	5.552879000	0.848894000
H	-11.112943000	4.793643000	0.008398000
H	-9.804371000	5.557711000	-0.917914000
N	7.139507000	0.000004000	-0.000063000
C	5.728085000	0.000018000	-0.000066000
C	5.015374000	0.965873000	0.740030000
H	5.553813000	1.701615000	1.327115000
C	3.630283000	0.954998000	0.739470000
H	3.102736000	1.712433000	1.309700000
C	2.899676000	0.000008000	-0.000030000
C	3.630269000	-0.954964000	-0.739563000
H	3.102712000	-1.712397000	-1.309787000
C	5.015361000	-0.965829000	-0.740159000
H	5.553786000	-1.701565000	-1.327263000
C	1.422888000	-0.000010000	0.000002000
C	0.694071000	-0.416704000	-1.131123000
H	1.217848000	-0.703943000	-2.037516000
C	-0.694105000	-0.416729000	-1.131092000
H	-1.217911000	-0.703986000	-2.037463000
C	7.849207000	1.222955000	-0.009232000
C	7.393516000	2.304367000	-0.790637000
H	6.511160000	2.189468000	-1.411141000
C	8.107264000	3.493217000	-0.801494000
H	7.763039000	4.311569000	-1.427302000
C	9.278803000	3.654045000	-0.037943000
C	9.717565000	2.564301000	0.738944000
H	10.612635000	2.668195000	1.345725000
C	9.025121000	1.364076000	0.757357000
H	9.365248000	0.544517000	1.381151000
C	10.031124000	4.956543000	-0.032966000
H	9.749880000	5.559236000	0.841310000
H	11.112186000	4.794750000	0.023243000
H	9.814737000	5.551489000	-0.924601000
C	7.849164000	-1.222977000	0.009173000
C	9.025086000	-1.364203000	-0.757465000

H	9.365207000	-0.544696000	-1.381331000
C	9.717425000	-2.564429000	-0.739020000
H	10.612446000	-2.668434000	-1.345870000
C	9.278625000	-3.654143000	0.038009000
C	8.107137000	-3.493236000	0.801497000
H	7.762831000	-4.311547000	1.427294000
C	7.393438000	-2.304295000	0.790604000
H	6.511078000	-2.189355000	1.411095000
C	10.031432000	-4.956364000	0.033178000
H	9.761657000	-5.552866000	-0.848895000
H	11.113070000	-4.793556000	-0.008591000
H	9.804620000	-5.557608000	0.917907000

1^{2+}-T

C	1.421322000	-0.000345000	-0.000337000
C	0.694314000	0.351226000	-1.152967000
H	1.219475000	0.587993000	-2.073207000
C	-0.694940000	0.351642000	-1.152573000
H	-1.220535000	0.588763000	-2.072479000
C	5.726359000	-0.000664000	-0.000702000
C	5.015538000	0.945106000	-0.765814000
H	5.554653000	1.665399000	-1.371392000
C	3.629477000	0.935043000	-0.764171000
H	3.100734000	1.677231000	-1.353287000
C	2.900509000	-0.000611000	-0.000656000
C	3.629474000	-0.936418000	0.762680000
H	3.100737000	-1.678561000	1.351857000
C	5.015532000	-0.946544000	0.764298000
H	5.554597000	-1.666772000	1.370008000
C	7.848803000	-1.222672000	-0.004199000
C	9.028178000	-1.360541000	0.758014000
H	9.371055000	-0.538073000	1.376403000
C	9.715094000	-2.564278000	0.750339000
H	10.608546000	-2.667538000	1.359381000
C	9.269982000	-3.659331000	-0.014757000
C	8.091135000	-3.504144000	-0.769455000
H	7.735570000	-4.331228000	-1.377301000
C	7.382936000	-2.312723000	-0.768891000
H	6.493154000	-2.203909000	-1.379690000
C	10.044870000	-4.948072000	-0.044558000

H	10.799863000	-4.922140000	-0.842232000
H	9.394822000	-5.805722000	-0.240848000
H	10.575218000	-5.120296000	0.896708000
C	7.847261000	1.223299000	0.005673000
C	7.380410000	2.310953000	0.772734000
H	6.491900000	2.199107000	1.384839000
C	8.091007000	3.501232000	0.781059000
H	7.738291000	4.323965000	1.396341000
C	9.270298000	3.658866000	0.028124000
C	9.719544000	2.564000000	-0.735371000
H	10.620004000	2.665517000	-1.334476000
C	9.030415000	1.361893000	-0.750876000
H	9.378214000	0.538799000	-1.365646000
C	10.017669000	4.964022000	0.019823000
H	9.712142000	5.575701000	-0.840059000
H	11.097559000	4.807822000	-0.064076000
H	9.819690000	5.548154000	0.922979000
N	7.140927000	-0.000154000	-0.000079000
C	-1.421470000	0.000380000	0.000440000
C	-0.694497000	-0.351253000	1.153056000
H	-1.219713000	-0.588121000	2.073244000
C	0.694757000	-0.351549000	1.152690000
H	1.220297000	-0.588573000	2.072646000
C	-5.726405000	0.000666000	0.000804000
C	-5.015639000	-0.946484000	0.764218000
H	-5.554793000	-1.667894000	1.368435000
C	-3.629562000	-0.936347000	0.762677000
H	-3.100736000	-1.679504000	1.350501000
C	-2.900667000	0.000642000	0.000766000
C	-3.629576000	0.937780000	-0.760956000
H	-3.100767000	1.680897000	-1.348845000
C	-5.015651000	0.947955000	-0.762475000
H	-5.554768000	1.669288000	-1.366827000
C	-7.848832000	1.222634000	0.007768000
C	-9.028376000	1.362738000	-0.753789000
H	-9.371448000	0.542036000	-1.374414000
C	-9.715190000	2.566502000	-0.742586000
H	-10.608767000	2.671546000	-1.351138000
C	-9.269801000	3.659382000	0.025458000
C	-8.090772000	3.501997000	0.779420000
H	-7.734982000	4.327362000	1.389466000
C	-7.382675000	2.310528000	0.775363000
H	-6.492751000	2.199949000	1.385635000

C	-10.044569000	4.948100000	0.059021000
H	-10.799421000	4.920002000	0.856756000
H	-9.394413000	5.805141000	0.257593000
H	-10.575079000	5.123008000	-0.881660000
C	-7.847217000	-1.223363000	-0.009281000
C	-7.379854000	-2.308782000	-0.779033000
H	-6.491058000	-2.195187000	-1.390395000
C	-8.090262000	-3.499270000	-0.790904000
H	-7.737019000	-4.320279000	-1.408146000
C	-9.269813000	-3.659125000	-0.039122000
C	-9.719590000	-2.566352000	0.727341000
H	-10.620223000	-2.669743000	1.325903000
C	-9.030755000	-1.364271000	0.746450000
H	-9.378858000	-0.542947000	1.363412000
C	-10.018127000	-4.963759000	-0.034823000
H	-9.738513000	-5.563880000	0.841798000
H	-11.099905000	-4.805346000	0.016520000
H	-9.795847000	-5.559841000	-0.924270000
N	-7.141040000	0.000116000	0.000139000
